# Skeletal Muscle Subpopulation Rearrangements upon Rhabdomyosarcoma Development through Single-Cell Mass Cytometry

**DOI:** 10.3390/jcm10040823

**Published:** 2021-02-17

**Authors:** Lucia Lisa Petrilli, Federica Riccio, Giulio Giuliani, Alessandro Palma, Cesare Gargioli, Simone Vumbaca, Monika Faron, Graziana Palmieri, Luca Pasquini, Francesca Sacco, Gianni Cesareni, Luisa Castagnoli, Claudia Fuoco

**Affiliations:** 1 Department of Biology, University of Rome “Tor Vergata”, 00133 Rome, Italy; lucialisa.petrilli@opbg.net (L.L.P.); federicariccio1989@libero.it (F.R.); g.giulianigiulio@gmail.com (G.G.); alessandro.palma@live.it (A.P.); Cesare.Gargioli@uniroma2.it (C.G.); simonevum41@gmail.com (S.V.); faron.monika@yahoo.it (M.F.); grazianapalmieri@gmail.com (G.P.); francesca.sacco@uniroma2.it (F.S.); 2 Department of Onco-Hematology, Cell and Gene Therapy, Ospedale Pediatrico Bambino Gesù (IRCCS), 00146 Rome, Italy; 3 Core Facilities, Istituto Superiore di Sanità, 00161 Rome, Italy; luca.pasquini@iss.it; 4 Fondazione Santa Lucia Istituto di Ricovero e Cura a Carattere Scientifico (IRCCS), 00143 Rome, Italy

**Keywords:** embryonal rhabdomyosarcoma, skeletal muscle, muscle populations, single cell, CyTOF, mass cytometry, tumor heterogeneity, mesenchymal cells, stem cells

## Abstract

The embryonal rhabdomyosarcoma (eRMS) is a soft tissue sarcoma commonly affecting the head and neck, the extremities and the genitourinary tract. To contribute to revealing the cell types that may originate this tumor, we exploited mass cytometry, a single-cell technique that, by using heavy-metal-tagged antibodies, allows the accurate monitoring of the changes occurring in the mononuclear cell composition of skeletal muscle tissue during tumor development. To this end, we compared cell populations of healthy muscles with those from spatiotemporal-induced eRMS tumors in a mouse model (LSL-Kras^G12D/+^;Tp53^Fl/Fl^) that can be used to develop rhabdomyosarcoma by means of infection with an adenovirus vector expressing Cre (Ad-Cre) recombinase. By monitoring different time points after tumor induction, we were able to analyze tumor progression and composition, identifying fibro/adipogenic progenitors (FAPs) as the cell type that, in this model system, had a pivotal role in tumor development. In vitro studies highlighted that both FAPs and satellite cells (SCs), upon infection with the Ad-Cre, acquired the potential to develop rhabdomyosarcomas when transplanted into immunocompromised mice. However, only infected FAPs had an antigen profile that was similar to embryonal rhabdomyosarcoma cells. Overall, our analysis supports the involvement of FAPs in eRMS development.

## 1. Introduction

The embryonal rhabdomyosarcoma (eRMS) is the most frequent highly malignant soft-tissue sarcoma in pediatric patients, accounting for 60% to 70% of RMS cases [1]. Because of their mesenchymal origin, eRMSs can emerge everywhere in the body, even where striated muscle is not normally found, such as the head, the neck region and the eye orbit [2]. However, even if eRMS can occur in body compartments relatively poor of skeletal muscle, it has a close resemblance to myogenic tissue. In fact, eRMS cells are characterized by a spindle shape [3], and occasionally are cross-striated and multinucleated like skeletal muscle fibers.

Most importantly, they recapitulate different steps of the myogenesis process by simultaneously expressing the early myogenic transcription factor Pax7 [4] together with late myogenic regulators, such as myogenin (Myog) and myoblast determination protein 1 (MyoD1) [5,6] as well as the markers of muscle differentiated cells desmin (Des) and α-smooth muscle actin (α-SMA) [6]. These observations lead to the proposal that eRMS tumors may originate from skeletal-muscle progenitors that fail to exit the cell cycle and undergo terminal differentiation. At the same time, eRMS cells would be blocked in a proliferating myoblast state, sharing with cancer cells stemness properties, such as self-renewal, high proliferation rate and resistance to senescence. Consistently, the genetic manipulation of myogenic progenitors in different phases of the differentiation process triggers eRMS onset frequently [7,8,9].

However, it was recently shown that cell types different from myogenic progenitors, such as endothelial or adipogenic precursor cells, could also induce eRMS formation, thus suggesting that the acquisition of muscle characteristics could be a consequence of a transdifferentiation triggered by tumor transformation [9,10].

Overall, these findings indicate that the eRMS cell composition and initiating cell type(s) still need to be clarified and may be diverse and dependent on the tumor model. Moreover, eRMSs, like many other solid tumors, are heterogeneous since they are formed by different subpopulations with distinct phenotypic features. This heterogeneity is a consequence of morphological and epigenetic plasticity, in addition to the coexistence of genetically divergent cell clones within tumors [10]. Thus, understanding eRMS heterogeneity would shed light on tumor development, possibly providing new clinically relevant insights on prognosis, resistance to chemotherapy and propensity to form metastasis.

In the work presented here, we characterized the tumorigenic potential of two cell populations represented by myogenic precursors known as satellite cells (SCs) and fibro/adipogenic progenitors (FAPs). Even if SCs are committed myogenic precursors, they can also enter an alternative mesenchymal differentiation (MAD) program, culminating in the formation of adipocytes [11,12,13,14]. FAPs are multipotent mesenchymal cells that provide trophic support to myogenic progenitor differentiation, thus promoting muscle-fiber specification and maturation [15,16,17,18]. However, FAPs are also the major cellular source of fibrosis in degenerative diseases, and are held responsible for heterotopic ossification in skeletal muscle [19,20].

To elucidate the role of such populations in eRMS biology, we adopted a spatially and temporally controlled mouse model (LSL-Kras^G12D/+^;Tp53^Fl/Fl^) and used single-cell mass cytometry to perform in vivo and in vitro multiparametric analyses [21].

## 2. Materials and Methods

### 2.1. Mouse Strains and Animal Procedures

The B6.129P2-Tp53tm1Brn/J (p53LoxP, RRID:IMSR_JAX:008462) mice were purchased from Jackson Laboratories, while the B6.129S4-Krastm4Tyj/J (LSL-KrasG12D, RRID:IMSR_JAX:008179) mice were kindly provided by Prof. F. Cecconi.

The LSL-Kras^G12D/+^;Tp53^+/+^ mice were crossed with the LSL-Kras^+/+^;Tp53^Fl/Fl^ mice. The resulting LSL-Kras^G12D/+^; Tp53^Fl/+^ mice were crossed with the LSL-Kras^+/+^;Tp53^Fl/Fl^ mice in order to generate the LSL-Kras^G12D/+^;Tp53^Fl/Fl^ mice that were used as a model for eRMS tumor induction.

Soft-tissue sarcoma were generated in the LSL-Kras^G12D/+^;Tp53^Fl/Fl^ mice by intramuscularly injecting a solution containing 45 µL of Opti-MEM (Gibco, Thermo Fisher Scientific, Monza, Italy, #31985070), 5 µL of the Ad-Cre (2 × 10^7^ pfu/mL, Vector Biolabs, Great Valley Parkway Malvern, USA, #1700) and 0.3 µL of CaCl_2_ 2 M. The infection was also introduced in mice of the same genetic background and age by using an empty-Ad virus only carrying the GFP reporter (Ctrl). Mice were sacrificed seven weeks from the tumor induction considering the time points of three, five and seven weeks after infection (three mice per time point). As we preferred to process the tumor samples at the same time, we first infected the mice to be analyzed after seven weeks and then, after two and four weeks, the mice corresponding to the five and three week time points, respectively.

The mice were bred respecting the standard animal facility procedures, and all the procedures were conducted in accordance with rules of good animal experimentation I.A.C.U.C. n°432 of March 12, 2006 and under ethical approval released on 23 October 2017 by the Italian Ministry of Health, protocol #820/2017-PR.

### 2.2. In Vivo Fluorescence Molecular Tomography

Fluorescence molecular tomography (FMT) was performed by using a Kodak FX image station 4000 (Kodak, New York, NY, USA) capable of acquiring transillumination, reflectance and absorption data.

Twenty-four hours before imaging, mice were injected intravitreal (I.V.) with 2 nmol/150 µL of a cathepsin activatable probe (Prosense 680, VisEn Medical, PerkinElmer, Italy) with an excitation wavelength of 680 nm and an emission wavelength of 720 nm. Before imaging, all mice were anesthetized with an intramuscular injection of saline solution containing ketamine (5 mg/mL) and xylazine (1 mg/mL) and shaved to reduce the influence of the fur on the optical signal.

At different chosen time points, the mice were positioned ventrally on the stage of the image station and noninvasively imaged by combining X-rays and fluorescence in the NIR (near infrared) region. The images were merged to obtain fusion images showing the signals of the activated cathepsins, as well as the anatomical structure of the tumor.

### 2.3. Histological Analysis

*Tibialis anterior* (TA) muscles were collected, embedded in an optimal cutting temperature compound (Killik-O.C.T., Bio Optica, Milan, Italy) and snap-frozen in liquid nitrogen for 10 s. Embedded muscles were stored at –80 °C for transverse cryosectioning with a Leica cryostat (Wetzlar, Germany). Cryosections (10 μm thickness) were collected on Superfrost glass slides (Thermo Fisher Scientific, Monza, Italy) and tissue slides were stained with hematoxylin and eosin (H&E). For the H&E staining, cryosections were fixed with 4% paraformaldehyde (PFA) for 15 min at room temperature (RT). After washing in pure water, cryosections were incubated in hematoxylin solution for 15 min and rinsed for 5 min in tap water. Cryosections were then counterstained with an alcoholic solution of eosin for 30 min. Following the eosin staining, cryosections were ethanol-dehydrated (one wash in 95% followed by three washes in 100%), clarified with the Histo-Clear solution (Agar Scientific, Stansted, UK) and finally mounted with coverslips using the resinous Eukitt mounting medium (Electron Microscopy Sciences, PA, USA #15320).

H&E images were captured using a Zeiss Lab A1 AX10 microscope (Carl Zeiss Microscopy, Oberkochen, Germany) at 40× magnification in brightfield.

### 2.4. Immunofluorescence

For Caveolin-3 (BD Transduction Laboratories, US, #610420) and α-SMA (α-smooth muscle actin) (Sigma-Aldrich, Merck, Darmstadt, Germany, #A5228) immunofluorescence staining, sections were fixed with 4% PFA for 10 min at RT, washed twice with 1X PBS and permeabilized with 0.3% Triton X-100 in 1X PBS for 30 min at RT. Unspecific binding sites were blocked for 1 h at RT with 10% normal goat serum, 1% glycine, and 0.1% Triton X-100 in 1X PBS.

Anti-Caveolin-3 and α-SMA primary antibodies were diluted 1:1000 and 1:100, respectively, in the blocking solution and incubated 1 h at RT. Sections were washed twice with a washing solution (1% BSA, 0.2% Triton X-100 in 1X PBS) and incubated for 30 min at RT with host-specific secondary antibodies. Finally, sections were washed twice with the washing solution and counterstained with 2 µg/µL Hoechst 33342 (Thermo Fisher Scientific, Monza, Italy #H3570) in PBS 0.1% Triton X-100 for 5 min at RT. Sections were washed twice with 1X PBS, mounted with Aqua-PolyMount (Polysciences, Germany) mounting medium and stored at 4 °C until further use.

### 2.5. Muscle Mononuclear and eRMS Cell Purification

Mice were sacrificed by cervical dislocation and the hind limbs were washed with 70% ethanol. For the isolation of single cells, muscle and tumor tissues were dissociated by following the same protocol. Briefly, mice hind limbs were dissected and finely mechanically minced in Hank’s balanced salt solution with calcium and magnesium (HBSS Gibco) supplemented with 0.2% bovine serum albumin (BSA) (AppliChem, Milan, Italy) and 1% penicillin–streptomycin (P/S) (Thermo Fisher Scientific, Monza, Italy, 10,000 U/mL) (HBSS^+^) under a sterile hood.

The homogenous tissue preparation was centrifuged at 700× *g* for 10 min at 4 °C to separate eventual fat pieces and subjected to an enzymatic digestion for 1 h at 37 °C, in gentle shaking, performed by resuspending the minced tissue into an enzymatic mixture containing 2 µg/µL collagenase A (Roche), 2.4 U/mL dispase II (Roche, Merck, Darmstadt, Germany) and 10 µg/mL DNase I (Roche) diluted in Dulbecco’s phosphate buffered saline (D-PBS) with calcium and magnesium (Gibco, Thermo Fisher Scientific, Monza, Italy).

Once digested, the enzymatic reaction was stopped with HBSS^+^ and the resulting cell suspension was subjected to three sequential filtrations through 100 µm, 70 µm and 40 μm cell strainers (BD Falcon, Milan, Italy) and centrifugations at 700× *g* for 5 min.

Red blood cell lysis was performed through incubation with RBC lysis buffer (Santa Cruz Biotechnology, D.B.A. Italia S.r.l., Segrate Milan, Italy) for 150 s in ice.

For magnetic-activated cell sorting (MACS, Miltenyi Biotech, Bologna, Italy) of muscle-cell populations derived from LSL-Kras^G12D/+^;Tp53^Fl/Fl^, pellets were resuspended in 500 µL of magnetic bead buffer (MBB) composed of 0.5% BSA and 2 mM EDTA in 1X PBS. Cell suspensions were filtered through a 30 µm preseparation filter (Miltenyi) and the mononuclear cells counted and centrifuged at 700× *g* for 10 min at 4 °C.

The cell populations of interest were separated with MS columns (Miltenyi 130-042-201) and selected with magnetic bead-conjugated antibody staining according to manufacturer’s instructions.

Antibody staining (CD45, CD31) was performed to identify nonmyogenic cells that were depleted using magnetic beads through the magnetic field. Among the CD45^−^/CD31^−^ (Lin^−^) cells, SCs were selected through α7-Integrin antibody expression, while FAPs were obtained by positive selection for Anti-Sca1 (non-HSC) antibody on the Lin^−^/α 7-Integrin^−^ cells.

### 2.6. Flow Cytometry

The purity of sorted cells was monitored by flow cytometry. A total of 1 × 10^5^ cells were incubated with primary antibodies on ice for 30 min in PBS containing 2 mM EDTA and 2% BSA. The following antibodies were used at specific dilutions: anti-α7-Integrin APC 1:500 (Ablab, Vancouver, Canada, #67001005), anti-Sca1 FITC 1:50 (BD Pharmingen, Milan, Italy, #557405), anti-CD31 PE 1:100 (BD Pharmingen #553373) and anti-CD45 eFluor 450 1:50 (eBioscience, Thermo Fisher Scientific, Monza, Italy, #48-0451-80).

Cell suspensions were washed with 2 mL of 2 mM EDTA and 2% BSA in 1X PBS and centrifuged at 4 °C for 10 min at 700× *g*. Unstained samples were prepared as control. Pellets were then resuspended in 100 µL of 2 mM EDTA and 2% BSA in 1X PBS and run to a flow cytometer. Approximately 10,000 events per sample were acquired with a CytoFLEX S (Beckman Coulter, Milan, Italy) instrument equipped with three lasers (488 nm, 405 nm and 638 nm) and 13 detectors. Quality control of the cytometer was assessed daily using CytoFLEX Daily QC Fluorospheres (Beckman Coulter #B53230). Data were collected by CytExpert 2.2 version (Beckman Coulter) software. If needed, a compensation matrix was calculated using a VersaComp Antibody Capture Kit (Beckman Coulter #B22804) according to the manufacturer’s instructions. FCS files were analyzed using CytExpert version 2.2 software.

### 2.7. Cell Culture

Freshly isolated eRMS cells and LSL-Kras^G12D/+^;Tp53^Fl/Fl^ FAP cells were seeded in growth medium composed of Dulbecco’s Modified Eagle Medium, High Glucose, GlutaMAX^TM^ (Gibco #61965-026) supplemented with 20% fetal bovine serum (FBS) (Euroclone, Milan, Italy, #ECS0180L), 100 U/mL penicillin and 100 mg/mL streptomycin (Gibco #15140122), 1 mM sodium pyruvate (Sigma-Aldrich #S8636) and 10 mM 4-(2-hydroxyethyl)-1-piperazineethanesulfonic acid (HEPES) (Sigma-Aldrich #H0887).

LSL-Kras^G12D/+^;Tp53^Fl/Fl^ SCs were cultured in a growth medium composed of Dulbecco’s Modified Eagle Medium, High Glucose, GlutaMAX^TM^, 100 U/mL penicillin and 100 mg/mL streptomycin, 1 mM sodium pyruvate, 10 mM HEPES, 20% FBS, 10% horse serum (Euroclone #ECS0090D) and 2% chicken embryo extract (Seralabs, Bergamo, Italy, #CE-650-J).

Once the isolated cells recovered their vitality, they were cultured and infected in Cytogrow (Resnova, Roma, Italy #TGM-9001-B) medium and expanded for several passages (more than P20).

### 2.8. In Vitro Infection of Sorted Cells

After cell sorting, 5 × 10^4^ cells were plated in a 35 mm dish and kept in culture condition as previously described, to allow the recovery of the cells. SCs and FAPs were infected within 4 days after magnetic sorting with a solution containing 750 µL of growth medium, 2 µL of the Ad-Cre virus (2 × 10^7^ pfu/mL, Vector Biolabs, #1700) and 0.5 µL of Polybrene (10 mg/mL Santa Cruz sc-134220) for 24 h.

Morphological changes were evaluated by acquiring subsequent images of the control cells (Ctrl), infected with the empty vector Ad-Ctrl and of the infected Ad-Cre SCs and Ad-Cre FAPs. They were compared at specific time points (one day, four days and seven days) after infection. Images were captured using a Zeiss Lab A1 AX10 microscope at the 20× magnification in brightfield.

For the proliferation assay, the number of the cells was evaluated by counting the cellular bodies in the acquired field at the different time points.

### 2.9. RNA Extraction, Retro-Transcription and Real-Time PCR

Ad-Cre SCs and Ad-Cre FAPs and Ctrl cells were washed in PBS and lysed in TRIzol^TM^ Reagent (Thermo Fisher Scientific, Monza, Italy #15596-018). Isolation of total RNA was performed according to manufacturer’s instructions.

RNA precipitation was performed at –20 °C overnight with glycogen. Total RNA was resuspended in nuclease-free water; the concentration and 260/280 nm ratio were determined with a Nanodrop Lite Spectrophotometer (Thermo Fisher Scientific). Samples were diluted to the concentration of 50 ng/µL and stored at –80 °C.

cDNA was generated with PrimeScript RT Reagent Kit (Takara #RR037A).

Real-time PCR was performed on 15 ng of cDNA using SYBR Premix Ex Taq (Tli RNaseH Plus) (Takara, Diatech Lab Line Srl, #RR420W) in a reaction volume of 20 µL. Tubulin was used as housekeeping gene. The following primers were used: *Tubulin* forward 5′-AAGCAGCAACCATGCGTGA-3′; *Tubulin* reverse 5′-CCTCCCCCAATGGTCTTGTC-3′; *Tp53* forward 5′-CACGTACTCTCCTCCCCTCAAT-3′; *Tp53* reverse 5′-AACTGCACAGGGCACGTCTT -3′. Comparative expression was performed using the 2^−ΔΔCt^ method [22].

### 2.10. Sphere Formation Assay

Ad-Cre SCs and Ad-Cre FAPs (2 × 10^4^) and eRMS cells were seeded in 6-well ultralow attachment microplates (Corning, USA) for 7 days. A DMEM/F12 serum-free medium (Invitrogen, USA #11330057) containing 5 μg/mL insulin, 20 ng/mL epidermal growth factor (EGF), 2% B27 and 20 ng/mL basic fibroblast growth factor (bFGF) was used to culture the spheres.

The total number of tumor spheres per plate was counted and images were acquired using an inverted microscope (Nikon, Amsterdam, Netherlands, model Eclipse Ts2 #136710).

### 2.11. Tumor-Induction Assay

Ad-Cre SCs and Ad-Cre FAPs were grown and then harvested from culture within 1-2 passages (P1-P2). Cells were washed and injected at defined numbers (1 × 10^5^) into the *tibialis anterior*, *gastrocnemius* and *quadriceps* muscles of three-month-old NOD/SCID mice previously anesthetized as already described. Mice were monitored for up to 3.5 weeks following cell transplantation for the emergence of tumors.

### 2.12. Single-Cell Mass Cytometry

For single-cell analysis via CyTOF2, 3 × 10^6^ cells from three independent mice for each condition were used. Cells were centrifuged at 600× *g* for 5 min and washed in D-PBS w/o calcium and magnesium (BioWest, Milan, Italy).

To minimize intersample staining variation, we applied mass-tag barcoding protocol on fixed cells. Cells were fixed with 1 mL Fix I buffer and incubated for 10 min at RT. The fixation was quenched with Barcode Perm Buffer (Fluidigm, CA, USA).

The samples from the different conditions were barcoded by individually incubating cells with the appropriate combination of palladium isotopes from the Cell-ID^TM^ 20-Plex Pd Barcoding Kit in Barcode Perm Buffer for 30 min at RT. The staining was quenched with MaxPar Cell Staining Buffer (Fluidigm, CA, USA). 

The antibody staining with metal-tagged antibodies against surface and intracellular antigens was performed on pooled samples after mass-tag cellular barcoding. Samples were collected into one unique tube, and the surface antibody-staining protocol was performed according to the manufacturer’s instructions for 30 min at RT. Surface-stained cells were then washed twice with MaxPar Cell Staining Buffer and permeabilized with ice-cold methanol on ice for 10 min. Membrane-permeabilized cells were washed twice with MaxPar Cell Staining Buffer and incubated with antibodies against intracellular antigens for 30 min at RT according to the manufacturer’s instructions.

The full list of antibodies is provided in Table 1.

After intracellular antibody staining, cells were washed twice with MaxPar Cell Staining Buffer and stained for 1 h at RT with an intercalation solution composed of Cell-ID Intercalator-Ir (191Ir and 193 Ir) (Fluidigm, CA, USA) into MaxPar Fix and Perm Buffer at a final concentration of 125 nM. Cells were washed twice with MaxPar Cell Staining Buffer and MaxPar Water supplemented with 0.1% Tween, respectively.

For mass-cytometry acquisition, cells were resuspended at the final concentration of 2.5 × 10^5^ cells per ml in ddH_2_O containing 10% of EQTM Four Element Calibration Beads and filtered through a 30-µm filter-cap FACS tube.

All the reagents and antibodies for mass cytometry experiments were purchased from Fluidigm Corporation (Fluidigm, CA, USA).

Samples were kept on ice prior to acquisition by using the mass-cytometry platform CyTOF2 (Fluidigm, CA, USA); data were collected as .fcs files.

### 2.13. CyTOF Data Processing

Following data acquisition, channel intensity was normalized using calibration beads [23] and files were debarcoded by using the Debarcoder software (Fluidigm). Data were gated using Cytobank (Beckman Coulter, Milan, Italy) [24].

Cells were identified by the incorporation of two iridium isotopes, 191Ir (DNA1) and 193Ir (DNA2). Next, singlets were discriminated from doublets using the event length parameter and analyzed using the tSNE [25,26] and FlowSOM [27] algorithms.

The viSNE maps in Figure 2 were generated by the tSNE algorithm with the following settings: 350,000 events using a proportional event sampling; 1000 iterations; perplexity 30; seed 641220519. The clustering channels were: CD146, CD34, F4/80, CD45, CD140α, CD25, CD140β, Vimentin, CD90.2, Cxcr4, α7-Integrin, Sca1, CD31, CD117, CD206, CD4 and Pan-Actin.

The FlowSOM algorithm was applied to define 20 metaclusters (Supplementary Figure S1A) sampling total events and using the following parameters: 10 iterations; 100 clusters; hierarchical consensus clustering to determine metaclusters; seed 1455244506; no scale normalization. The same clustering channels of the tSNE algorithm were applied in the FlowSOM analysis. By consulting the viSNE maps in Supplementary Figure S1B, we merged metaclusters with a similar expression profile to obtain 7 metaclusters corresponding to the main cell populations of the skeletal muscle. Finally, such metaclusters were mapped onto the viSNE maps to facilitate result interpretation (Figure 2C). We selected the same clustering channels applied for the tSNE algorithm.

The Heatmap in Figure 2B was generated by exporting the mean intensity values from Cytobank and by normalizing these values between –2 and 2 using the R environment.

The FlowSOM algorithm in Figure 4 was applied to define 30 metaclusters sampling total events and using the following parameters: 1000 iterations; 196 clusters; hierarchical consensus clustering to determine metaclusters; perplexity: 30 seed 167091349; no scale normalization. By consulting the viSNE maps in Supplementary Figure S4, we merged the metaclusters with a similar expression profile to obtain 9 metaclusters corresponding the main cell populations of the skeletal muscle. The clustering channels were: α7-Integrin, CD140α, CD140β, CD34, CD90.2, Cxcr4, Sca1 and Vimentin. The heatmap presenting the results of this experiment (Figure 4B) was generated by exporting the mean intensity value from Cytobank to the R environment, as explained.

The viSNE maps in Figure 5 were generated by running the tSNE algorithm with the following settings on the total available events: 1000 iterations; perplexity 30; seed 1535857729. The clustering channels were: CD146, CD34, CD140α, CD140β, Vimentin, CD90.2, α7-Integrin and Sca1.

The multidimensional scaling (MDS) illustrated in Figure 5B was performed on the .fcs files by using the R Limma package (Melbourne, Australia) [28].

### 2.14. Statistical Analysis

Data were from at least 3 independent samples unless otherwise indicated. Results are presented as means ± SEM. Statistical evaluation was conducted by using the one-way and two-way ANOVA as indicated in the figure legend. Comparisons were considered statistically significant at * *p* < 0.05; ** *p* < 0.01; *** *p* < 0.001; **** *p* < 0.0001.

All statistical analysis was performed using Prism 6 (GraphPad, CA, USA).

## 3. Results

### 3.1. Generation of eRMS in LSL-Kras^G12D/+^;Tp53^Fl/Fl^ Mice

We first explored the timing of the histological and cellular modifications occurring in the skeletal muscle after eRMS onset. To this end, we adopted a genetically engineered mouse model carrying conditional oncogenic Kras (G12D) and Tp53 knockout alleles (LSL-Kras^G12D/+^;Tp53^Fl/Fl^) [21].

**Figure 1:**
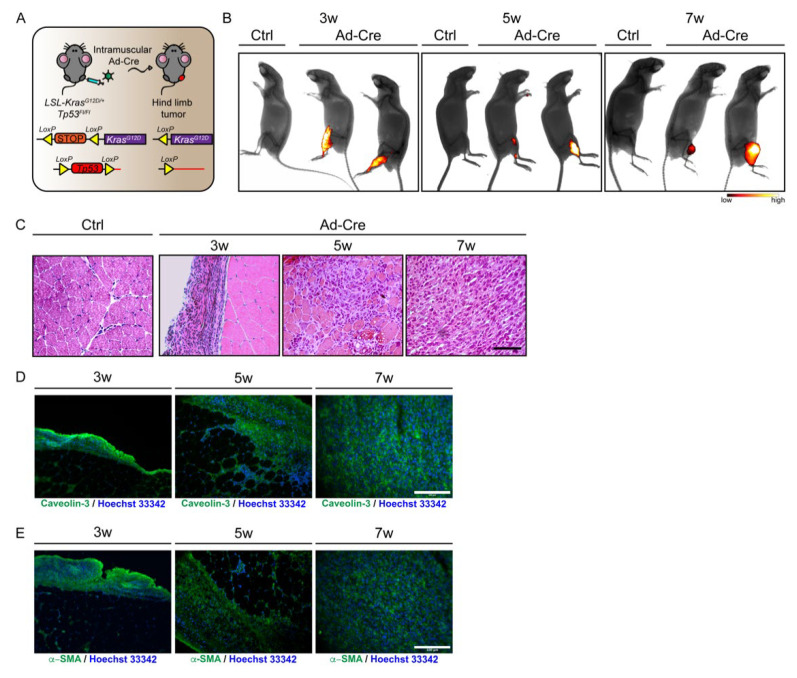
Generation of eRMS tumors in LSL-Kras^G12D/+^;Tp53^Fl/Fl^ mice. (**A**) Experimental workflow. LSL-Kras^G12D/+^;Tp53^Fl/Fl^ conditional mice were injected intramuscularly with Ad-Cre in order to induce genetic rearrangements, the constitutive activation of Kras and the inactivation of Tp53, which lead to eRMS tumor masses in a spatially and temporally controlled manner. (**B**) LSL-Kras^G12D/+^;Tp53^Fl/Fl^ conditional mice were injected with Ad-Cre and Ctrl vector. Tumor growth was assessed at three, five and seven weeks after Ad-Cre infection through FMT performed by intravitreal administration of the fluorescent probe (ProSense 680). This probe was activated by tumor cathepsin proteases to yield a fluorescence signal (black: low cathepsin activity; white: high cathepsin activity). (**C**) Representative H&E staining on histological sections from Ctrl and Ad-Cre infected muscles at three, five and seven weeks after Ad infection. (**D**,**E**) Immunofluorescence staining on sections of Ad-Cre infected muscles at three, five and seven weeks after infection. Sections were stained with antibodies raised against Caveolin-3 (**D**) and α-SMA (**E**). Scale bars: 100 µm.

This model allows limiting of the induction of the oncogenic mutations to the tissue of interest at a specific time. Hence, the model was obtained by injecting an adenovirus expressing Cre recombinase (Ad-Cre) into the hind limb muscles of 45-day-old mice, thus promoting the spatially and temporally controlled chromosomal rearrangements that activate the oncogenic mutations (Figure 1A).

In a preliminary analysis, we assessed the kinetics of tumor development in the hind limbs at three, five and seven weeks after Ad-Cre infection. Tumor progression was monitored by fluorescence molecular tomography (FMT). The emerging tumors were imaged by injecting a fluorescent probe (ProSense 680) that was specifically activated by cathepsin proteases, a tumor-specific enzymatic activity [29].

The FMT analysis showed the activation of cathepsin proteases at all the considered time points, including the very early time point of three weeks (Figure 1B). For this reason, the three time points (three, five and seven weeks) were all considered in the following experiments designed to observe the changes in the tissue histology and mononuclear cell composition of the growing tumors.

Consistent with the analysis of tumor growth by FMT, H&E staining revealed tissue alterations characterized by many interstitial cells already surrounding myofibers at the three-week time point. The observed modifications become more evident at the five-week time point. Seven weeks after Ad-Cre infection, the tumor was fully developed, highlighting major changes occurring between the fifth and seventh weeks. Indeed, after the five-week time point, the typical skeletal muscle tissue organization was no longer detectable, and spindle-shaped rhabdomyoblast cells, with high eosinophilic cytoplasm, had completely replaced myofibers in the seven-week tumor mass (Figure 1C).

In order to authenticate the eRMS nature of the generated tumor mass, we monitored two eRMS markers through immunofluorescence on tissue sections. This analysis highlighted a diffuse expression of Caveolin-3 (Figure 1D) and α-smooth muscle actin (α-SMA) (Figure 1E), both sensitive markers of eRMS, further confirming the generation of eRMS tumor in our mouse model [30].

These preliminary experiments validated the chosen model for a controlled and efficient induction of eRMS.

### 3.2. Immunophenotyping of Skeletal-Muscle Mononuclear Cell Populations upon eRMS Induction

Next, we aimed at characterizing at the single-cell level the phenotypic changes between the cell populations in the homeostatic muscle tissue and those observed in the tumor mass after tumor induction.

The hind limb muscles were collected from Ad-Cre infected mice and from Ctrl counterparts of the same genetic background. The separated tissue was enzymatically digested to isolate mononuclear cells, and after mass tag-barcoding [31], samples were pooled for antibody staining with a panel of 18 antibodies designed to cover the main markers of immune and skeletal muscle-cell populations.

**Figure 2:**
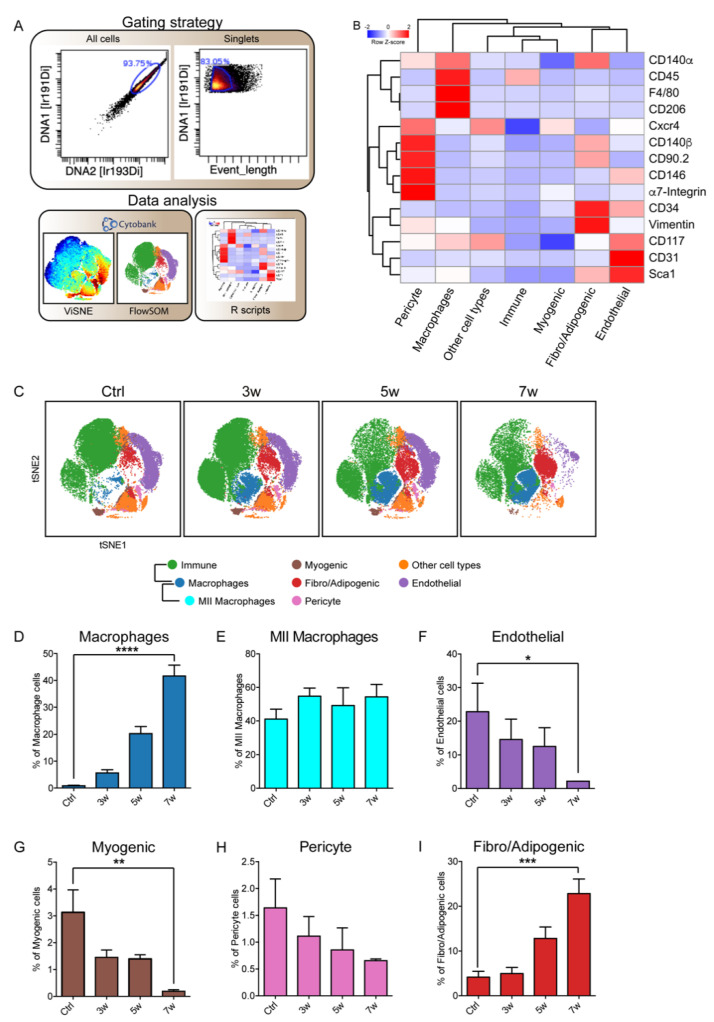
Mass cytometry analysis of skeletal muscle-cell subpopulations upon the induction of eRMS tumors in LSL-Kras^G12D/+^;Tp53^Fl/Fl^ mice. (**A**) Workflow of the gating strategy. Cells were manually gated from debris on the basis of DNA content monitored by the incorporation of the Ir intercalator. Doublets were then excluded according to the event-length parameter. Manually gated singlet events were used for tSNE and FlowSOM analysis in Cytobank. (**B**) FlowSOM heatmap of column-normalized (z-score) marker expression for each of the identified clusters. Colors varied according to the expression level of the considered marker in a blue-to-red scale, indicating low to high expression, respectively. (**C**) Time course of the variation in muscle-cell subpopulation abundance upon Ad-Cre infection. The dynamic changes are illustrated by representative density plots generated with FlowSOM algorithm. (**D**–**I**) The bar plots quantitate the variation in population relative abundance evaluated three, five and seven weeks after Ad-Cre hind-limb infection and compared with Ctrl mice. With the exception of the MII macrophage number determined on the total number of macrophages, cell percentages were assessed on the total number of cells in each sample (*n* = 3). The statistical significance was estimated by one-way ANOVA. All data are represented as mean ± SEM, and the statistical significance was defined as * *p* < 0.05; ** *p* < 0.01; *** *p* < 0.001; **** *p* < 0.0001.

The barcoded cell suspensions were analyzed by a single-run experiment with the CyTOF2 mass cytometer [32,33]. Signals were de-barcoded to assign events to the different samples, and single cells were identified by monitoring the DNA content and the event-length parameter (Figure 2A). Single cells were analyzed by applying the tSNE [25,26] and FlowSOM algorithms [27]. To better discern the FlowSOM output, we mapped the obtained metaclusters onto viSNE maps produced by applying the t-distributed stochastic neighbor embedding (tSNE) algorithm (Supplementary Figure S1). Thus, by looking at the antigenic profile of the FlowSOM’s 20 metaclusters, we mapped seven different cell types of muscle-resident populations onto viSNE maps (Figure 2B,C).

Cells of the immune compartment could be identified as they expressed the CD45 antigen. Within this large population, macrophages were recognized as they presented the F4/80 antigen, while the MII macrophage subpopulation also coexpressed CD206. Cell populations that did not express the CD45 antigen contained the remaining muscle mononuclear cell populations (Figure 2B).

In the latter, we identified a FAP-like population including cells coexpressing Sca1, CD90.2, Vimentin, CD140α and CD34 [15,34,35,36,37,38,39,40,41,42]. We also recognized myogenic-like cells that expressed α7-Integrin and Cxcr4 (C-x-c chemokine receptor type 4, also known as CD184) and a pericyte-like population characterized by CD146 and CD140β expression [37,43,44,45,46]. We also labeled a cluster as endothelial cells, since it contained cells expressing the two endothelial markers CD31 and CD117 [47,48,49,50]. The antigen profile of four minor additional clusters was also highlighted. They were dubbed as “other cell types” (Figure 2B), and their variation in time is shown in Supplementary Figure S2A,B.

The antibody panel that we used did not allow us to describe a newly arising population with a distinctive tumor-specific antigenic profile. However, major changes were observed in the relative abundance of the different populations that were identified.

Macrophages, which were a minor fraction of the CD45 population in control conditions, significantly (**** *p* < 0.0001) increased to reach their maximum seven weeks after tumor induction, while MII macrophages, expressing the CD206 antigen, did not change in number (Figure 2D,E). A significant (* *p* < 0.05) decrease was observed in the endothelial population, possibly paralleling an impairment of the tumor tissue’s vascular organization (Figure 2F). Consistent with the progressive disruption of the muscle myofiber organization, the myogenic α7-Integrin-expressing population also become less abundant (Figure 2G). Moreover, a similar albeit less important decrease was observed in the pericyte population (Figure 2H). In contrast with the observed decreasing trend of muscle-resident progenitor cells, the FAP-like population dramatically increased in size and comprised up to 23% of the total mononuclear cell population seven weeks after tumor induction (Figure 2I). Interestingly, this subpopulation was particularly enriched in Vimentin and CD90.2 coexpressing cells (Supplementary Figure S2C).

Thus, our high-dimensional analysis pointed to FAP-like cells as the main cell type populating seven-week eRMS tumor masses.

### 3.3. Skeletal-Muscle Progenitor Populations (FAPs and SCs) Isolated from LSL-Kras^G12D/+^;Tp53^Fl/Fl^ Mice Promote Tumor Transformation

We next asked whether progenitor cells derived from the LSL-Kras^G12D/+^;Tp53^Fl/Fl^ model could induce the formation of RMS after in vitro infection with the Ad-Cre vector and transplantation into immunocompromised mouse muscles. We focused on SCs, considered the most likely origin of eRMS, and on FAPs, the population that best matched the eRMS immunophenotype at seven weeks in our model. SCs and FAPs were respectively purified as CD31^−^/CD45^−^/α7-Integrin^+^/Sca1^-^ and CD31^−^/CD45^−^/α7-Integrin^−^/Sca1^+^ cells from muscle mononuclear cells by using MACS microbead technology. The purity of the isolated cell populations was checked by flow cytometry (Supplementary Figure S3A).

**Figure 3:**
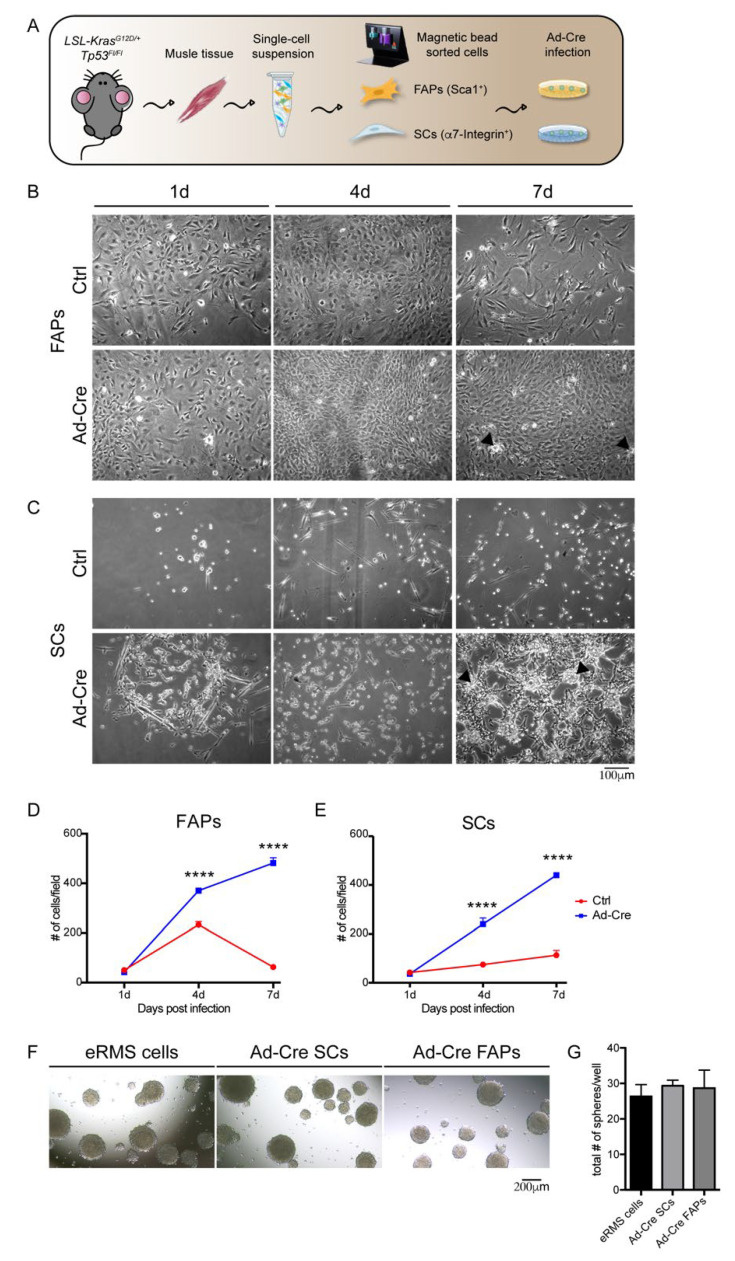
Analysis of the tumorigenic properties acquired by muscle-cell populations upon in vitro induction of genetic rearrangement by Ad-Cre infection. (**A**) Experimental workflow. FAPs and SCs magnetically sorted from LSL-Kras^G12D/+^; Tp53^Fl/Fl^ conditional mice were infected in vitro with Ad-Cre in order to induce the genetic rearrangements. (**B**,**C**) Representative images of Ad-Cre FAPs (**B**) and Ad-Cre SCs (**C**) together with Ctrl cells. Morphological changes between Ctrl and Ad-Cre cells were evaluated one, four and seven days (d) post-infection. Scale bar: 100 µm. (**D**,**E**) Growth curves of Ctrl and Ad-Cre FAPs (**D**) and Ad-Cre SCs (**E**). The total cell number (#) was defined by counting cellular bodies on brightfield images. The growth curves were derived from three independent biological replicates. Statistical analysis was performed by applying a two-way ANOVA test (**** *p* < 0.0001). (**F**) Rhabdo-sphere formation. Representative images of eRMS cells, Ad-Cre FAP- and Ad-Cre SC-generated spheres under low attachment conditions. Scale bar: 200 µm. (**G**) Quantification of rhabdo-sphere number. The number of the spheres generated by eRMS cells, Ad-Cre FAPs and Ad-Cre SCs was evaluated by counting the spheres present in each well of technical duplicates from three independent biological replicates.

To induce the genetic rearrangements leading to the activation of Kras(G12D) and inactivation of Tp53, cells were infected in vitro with the Ad-Cre (Figure 3A). The rearrangements induced by the Cre recombinase were confirmed by monitoring *Tp53* downregulation by real-time PCR, and Kras(G12D) constitutive activation by Western blot analysis (Supplementary Figure S3B,C) three days after infection.

The oncogenic effect of the induced genetic modifications was assessed by monitoring the changes in the morphology and proliferation of transformed cells at one, four and seven days after virus infection (1d, 4d, 7d) (Figure 3B–E). Ad-Cre FAPs and Ad-Cre SCs one day post-infection already showed a more robust proliferation when compared with the same cell types infected with the Ctrl virus. This difference became more evident at later time points, when the infected cell culture reached a higher confluence and formed foci (Figure 3B,C). Moreover, both Ad-Cre-infected cell types proliferated at a higher rate than their respective controls (Figure 3D,E). In particular, while the Ctrl FAPs already became senescent and the Ctrl SCs terminally differentiated into myotubes seven days post-infection, the Ad-Cre cells retained a higher proliferation potential for several in vitro passages (Supplementary Figure S3D).

The tumorigenic potential acquired by Ad-Cre FAPs and Ad-Cre SCs was further confirmed by evaluating their ability to form rhabdo-spheres when cultured under low-attachment conditions. After seven days, both Ad-Cre-infected cell types showed a tendency to aggregate and form spheroid structures (Figure 3F). This property is shared by primary eRMS cells isolated from the tumor mass generated in vivo and kept in the same culture conditions (Figure 3G).

To further test the in vivo oncogenic potential of Ad-Cre FAPs and Ad-Cre SCs, we separately inoculated them into the *tibialis anterior* muscles of NOD/SCID immunocompromised mice (Supplementary Figure S3E).

The intramuscular injection had already reproducibly generated palpable tumors three weeks after the inoculation (Supplementary Figure S3F). The histological analysis carried out on tissue sections after the inoculation of Ad-Cre FAPs and Ad-Cre SCs revealed that both the infected cell types were able to generate a tumor mass in all inoculated mice. The presence of dense areas of nuclei with frequent mitotic figures and a high cellular activity, together with the loss of the physiological organization of the muscle tissue, confirmed the transforming ability of the in vitro infected cells and the tumor-forming potential of both of these cell populations (Supplementary Figure S3G).

**Figure 4:**
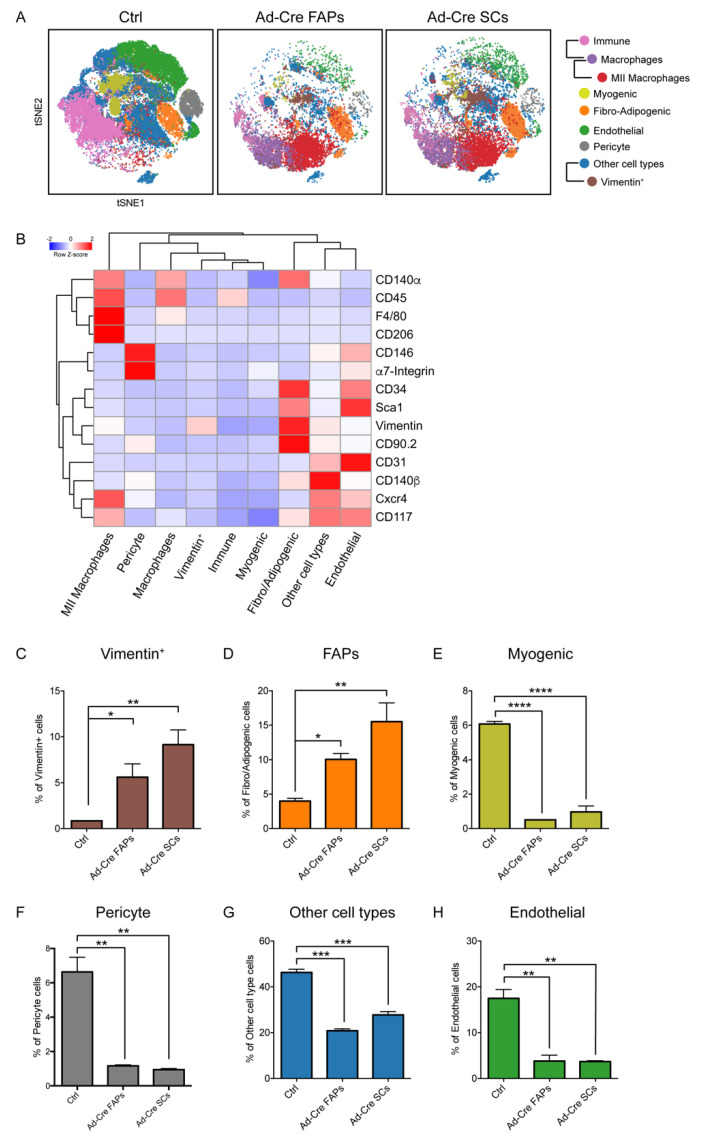
Mass-cytometry analysis of allograft tumors generated in NOD/SCID immunocompromised mice upon Ad-Cre FAP and Ad-Cre SC inoculation. (**A**) Variation in skeletal muscle-cell population abundance 3.5 weeks after the inoculation of Ad-Cre FAPs and Ad-Cre SCs into NOD/SCID mice. The dynamic changes are illustrated by representative density plots generated with the FlowSOM algorithm. (**B**) FlowSOM heatmap of column-normalized (z-score) marker expression for each of the identified clusters. Colors varied according to the expression level of the considered marker in a blue-to-red scale, indicating low to high expression, respectively. (**C**–**H**) The bar plots quantitate the variation in population abundance evaluated 3.5 weeks after cell inoculation. Cell percentages were assessed on the total number of cells in each sample (*n* = 2). The statistical significance was estimated by one-way ANOVA. All data are represented as mean ± SEM, and the statistical significance was defined as * *p* < 0.05; ** *p* < 0.001; *** *p* < 0.001; **** *p* < 0.0001.

We adopted a multiparametric approach as previously described (Figure 2 and Figure S1) to characterize the hind-limb muscles of the NOD/SCID mice inoculated with Ad-Cre FAPs and Ad-Cre SCs (Figure 4 and Figure S4). Tissues inoculated with Ad-Cre FAPs displayed a cell organization more similar to that observed in Ad-Cre SC-inoculated muscles rather than to the Ctrl (Figure 4A), suggesting that both Ad-Cre cell types behaved similarly after injection.

This was further confirmed by looking at the abundance of the cell populations in the nonimmune compartment, where little difference could be observed in mice inoculated with either infected cell type (Figure 4C–H). Most of the identified cell populations decreased in abundance after cell inoculation, the exception being the cell population expressing Vimentin and FAPs that became more numerous in inoculated mice (Figure 4C,D).

### 3.4. Immunophenotyping of the In Vitro-Transformed Progenitor Cells Emphasizes FAPs as Candidates eRMS Origin-Cell

Once we confirmed the oncogenic potential of both in vitro-transformed cell types, the immunophenotypic profiles of Ad-Cre FAPs and Ad-Cre SCs were compared to that of eRMS cells isolated from tumor masses in our mouse model system. We used mass cytometry in order to obtain hints about the progenitor cell populations that had the potential to give origin to eRMS.

To this purpose, tumors generated by in vivo infection of muscles from conditional LSL-Kras^G12D/+^;Tp53^Fl/Fl^ mice were surgically resected seven weeks after Ad-Cre in vivo infection and enzymatically digested to extract mononuclear cells. The isolated cells were kept in culture for five passages, as the in vitro infected Ad-Cre FAPs and Ad-Cre SCs, in order to select transformed cells and to establish tumor cell lines.

The cell samples were separately barcoded, combined and labeled with the previously optimized antibody panel (Table 1). The metal-tagged cell mixture was analyzed in a single-run experiment on a CyTOF2 mass cytometer. The single events were de-barcoded and analyzed by applying the tSNE algorithm.

**Figure 5:**
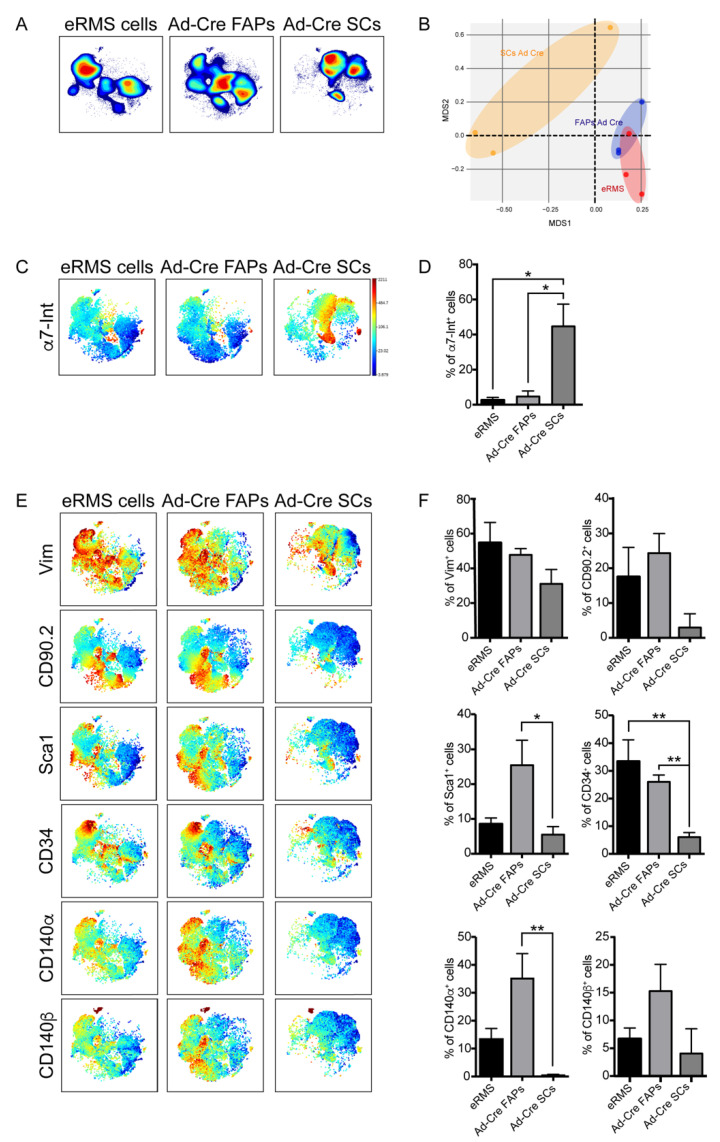
Mass cytometry antigenic-profile comparison of eRMS cells and Ad-Cre FAPs and Ad-Cre SCs. (**A**) Representative viSNE contour plots showing cell-density distribution of eRMS cells and Ad-Cre FAPs and Ad-Cre SCs (red: high density; blue: low density). (**B**) Multidimensional scaling plot (MDS) of eRMS, Ad-Cre FAPs and SCs. (**C**) Representative viSNE maps colored according to the expression level of α7-Integrin marker (blue: low expression; red: high expression). (**D**) The bar plots quantitate the percentage of cells positive for the expression of the α7-Integrin marker. (**E**) Representative viSNE maps showing the expression level of the indicated marker. (**F**) The bar plots quantitate the percentage of cells positive for the expression of the analyzed marker. (**D**,**F**) Cell percentages were assessed on the total number of cells in each sample (*n* = 3). The statistical significance was estimated by one-way ANOVA. All data are represented as mean ± SEM, and the statistical significance was defined as * *p* < 0.05; ** *p* < 0.01.

Cell-density analysis revealed the presence of different subpopulations among the primary eRMS cells whose spatial distribution was more similar to that of Ad-Cre FAPs (Figure 5A). This close similarity was also confirmed by a multidimensional scaling plot (MDS) (Figure 5B), which showed that the Ad-Cre FAPs were closer to eRMS cells than to Ad-Cre SCs in the two-dimensional space defined by MDS coordinates. The same conclusion was highlighted when comparing the bidimensional viSNE maps generated for the expression profiles of the eRMS subpopulations with those of Ad-Cre FAPs and Ad-Cre SCs (Figure 5C–F). In particular, eRMS subpopulations, as well as Ad-Cre FAPs, expressed very low levels of α7-Integrin (Figure 5C,D). On the contrary, they turned out to be positive for the expression of the cell-surface antigens Vimentin, CD90.2, Sca1, CD34, CD140α and CD140β, which are markers of mesenchymal cells. When comparing this expression profile with those of Ad-Cre FAPs and Ad-Cre SCs, we observed that only Ad-Cre FAPs expressed a similar expression pattern to most of the analyzed antigens (Figure 5E). To quantitatively describe these observations, we estimated the fraction of cells that expressed a specific antigen in each sample (Figure 5F and Figure S5A). It is interesting to note that the expression of CD31, a well-established marker of endothelial cells used to monitor the microvessel density (MVD) in malignant tissues [51,52], was mainly expressed only by eRMS and Ad-Cre FAPs, suggesting that Ad-Cre FAPs probably had a greater ability to sustain angiogenic processes during tumorigenesis (Supplementary Figure S5B,C). Moreover, the stem-cell marker CD117, commonly expressed in eRMS and generally in cancer cells, was more expressed by Ad-Cre FAPs in comparison to Ad-Cre SCs. Finally, Cxcr4, a common marker that together with α7-Integrin characterizes SCs and has a role in promoting tumor spread and metastasis [53], was also observed at similar levels in Ad-Cre FAPs and eRMS cells (Supplementary Figure S5B,C).

## 4. Discussion

The eRMS is a highly malignant pediatric soft-tissue sarcoma that is often associated to mutations in the *Tp53* gene and aberrant expression of RAS and skeletal muscle markers. The eRMS can develop in body compartments where striated muscle is not ordinarily found. This is probably due to a muscle lineage commitment activated in the eRMS cell of origin, regardless of the primary site of tumor onset.

We have used a systematic approach based on the in vivo and ex vivo genetic manipulation of distinct populations of muscle cells to investigate the developmental origin of eRMS.

A few reports have pointed to SCs as the main cell type involved in eRMS onset because of their gene expression signature. Consistently, eRMSs express markers of quiescent or activated satellite cells such as Pax7, MyoD and Myog that could contribute to tumor development [4,5,9]. Moreover, SCs also express the oncogene c-Met [54,55,56], which further links them to neoplastic transformation. Hettmer and colleagues demonstrated that SCs, if triggered by particular oncogenic lesions (activation of Kras(G12V) and deletion of p16INK4Ap19ARF), can generate murine sarcomas that are very similar to human nonalveolar RMS with pleomorphic features [57]. The tumorigenic potential of SCs in promoting eRMS was also investigated through a mouse model of Duchenne muscular dystrophy (DMD) lacking *Tp53* gene (Tp53^KO^mdx/mTR), demonstrating that the DMD severity reduced the latency of RMS formation and increased the ability of SCs to develop rhabdomyosarcomas when injected into immunodeficient mice [58].

On the other hand, other reports have implicated adipocytes or endothelial progenitors as eRMS initiating cells. These reports independently demonstrated the involvement of the Sonic Hedgehog (Shh) pathway in controlling eRMS development. In particular, it was found that the restricted activation of Shh into adipocytes or endothelial progenitor cells allowed them to generate rhabdomyosarcoma in the head and ventral neck region, the most common location of eRMS [59,60]. Moreover, mesenchymal stem cells have also been involved in malignant transformation, leading to the formation of many types of sarcoma, including rhabdomyosarcoma [61].

In order to shed light on the cellular origin of eRMS, we explored the involvement of skeletal-muscle populations in eRMS onset by applying a single-cell mass-cytometry approach. To recapitulate the genetic alterations leading to eRMS, we used a conditional eRMS mouse model (LSL-Kras^G12D/+^;Tp53^Fl/Fl^) in which we triggered the activation of the Kras(G12D) and the knockout of Tp53 proteins in a spatially and temporally controlled manner by infecting them with Ad-Cre [21,62]. RMS is the most frequently observed pediatric cancer in classic Li–Fraumeni cancer-syndrome families with germline mutations of the *Tp53* tumor suppressor gene [63].

In our in vivo system, major changes in skeletal muscle architecture became noticeable between three and seven weeks after Ad-Cre infection when a tumor mass expressing eRMS markers, such as α-SMA and Caveolin-3 [30], was observed.

By using a panel of 18 antibodies to discriminate the main cell types in the skeletal muscle-cell niche, we observed that eRMS onset paralleled a major shift in the abundance of the resident muscle-cell population profile. Interestingly, the main cell type observed in the nonimmune compartment seven weeks after tumor induction, when tumor masses were fully developed, was a fibro/adipogenic-like population characterized by the expression of the Sca1, CD90.2, Vimentin, CD140α and CD34 markers. This is consistent with the notion that eRMS cells express Vimentin and CD90.2, and that a subpopulation of soft tissue sarcoma cancer stem cells expressing CD140α exists [64].

To confirm the potential of mesenchymal cells to generate eRMS, we isolated FAP and SC cells from the LSL-Kras^G12D/+^;Tp53^Fl/Fl^ conditional mouse model and cultured them in vitro. Upon infection with Ad-Cre, both cell types acquired tumorigenic properties, as they showed a high proliferation rate and ability to form foci and rhabdo-spheres. However, when the antigen profiles of Ad-Cre FAPs and Ad-Cre SCs were compared to that of eRMS cells, isolated from Ad-Cre in vivo-generated tumor masses and in vitro cultured, we observed that only Ad-Cre FAPs expressed eRMS markers, even if both Ad-Cre FAPs and Ad-Cre SCs were able to induce tumor formation when implanted into NOD/SCID mice. Moreover, the mass-cytometry analysis performed on Ad-Cre FAP and Ad-Cre SC cell-derived tumors did not highlight any differences in terms of subpopulation composition of the generated tumors, suggesting that these populations may both contribute to tumor development. In fact, Rubin and colleagues demonstrated that eRMS and some undifferentiated pleomorphic sarcomas are a continuum of diseases, often with a similar mutational profile, but generally arising from different cell populations [7].

Two abundant subpopulations, aside from those of the immune compartment, were identified in the implanted mice. One of these subpopulations expressed the same markers of the fibro/adipogenic progenitors, while the other was dubbed “Vimentin^+^” since it was particularly enriched in the expression of this antigen, one of the main eRMS marker. These observations further support a mesenchymal nature of eRMS tumoral tissue, which is a common denominator for a wide group of sarcoma tumors. In particular, it has been suggested that the transformed mesenchymal stem cells can give rise to a particular subtype of sarcoma, depending on the vulnerability to mutations that affect specific developmental pathways [65].

Moreover, our analysis showed that myogenic progenitors, as well as other identified subpopulations, decreased upon Ad-Cre FAP and Ad-Cre SC inoculation into NOD/SCID mice. This suggests the possibility that other cell subpopulations, such as myogenic progenitors, for which an alternative mesenchymal pathway has already been described [14], could populate the tumor mesenchymal compartment through a transdifferentiation process.

Our observations emphasized the potential of mesenchymal cells to develop eRMS. However it remains to be clarified whether, in vivo, tumors may originate directly from a mesenchymal cell, or if a different cell type transdifferentiates into a more mesenchymal population that in turn forms the tumor.

## Figures and Tables

**Table 1:** List of the metal-tagged antibodies used in the mass cytometry experiments.

Antibody	Metal
Anti-mouse CD146	141Pr
Anti-mouse CD34	144Nd
Anti-mouse F4/80	146Nd
Anti-mouse CD45	147Sm
Anti-mouse CD140α	148Nd
Anti-mouse CD25 (Il-2R)	150Nd
Anti-mouse CD140β	151Eu
Anti-mouse CD274	153 Eu
Anti-Vimentin	154Sm
Anti-mouse CD90.2 (Thy-1.2)	156Gd
Anti-mouse Cxcr4 (CD184)	159Tb
Anti-mouse α7-Integrin	161Dy
Anti-mouse Sca1 (Ly-6A/E)	164Dy
Anti-CD31 (Pecam1)	165Ho
Anti-mouse CD117	166Er
Anti-mouse CD206	169Tm
Anti-mouse CD4	172Yb
Anti-Pan-Actin	175Lu

## Data Availability

All data generated or analyzed in this study are available upon reasonable request.

## Supplementary Materials

The following are available online at https://www.mdpi.com/2077-0383/10/4/823/s1: Figure S1: Mass-cytometry characterization of skeletal muscle cell subpopulations upon the induction of eRMS tumors in LSL-KrasG12D/+; Tp53Fl/Fl mice. Figure S2: Mass-cytometry characterization of “Other cell type” and of FAPs. Figure S3: Characterization of FAP and SC cells isolated from LSL-KrasG12D/+; Tp53Fl/Fl conditional mice and injected into NOD/SCID mice after in vitro infection with Ad-Cre virus. Figure S4: Mass-cytometry characterization of allograft tumors generated upon the inoculation of Ad-Cre FAPs and Ad-Cre SCs in NOD/SCID mice. Figure S5: Phenotypic comparison between Ad-Cre infected FAPs and Ad-Cre SCs and eRMS cells isolated from in vivo generated tumors.
