# Supplementary material for: Skeletal Muscle Subpopulation Rearrangements upon Rhabdomyosarcoma Development through Single-Cell Mass Cytometry
Source: J Clin Med. 2021 Feb 17;10(4):823. doi: 10.3390/jcm10040823 (PMC7922544; doi:10.3390/jcm10040823)
Supplement: Supplementary file 1 [file jcm-10-00823-s001.pdf]

Supplementary material

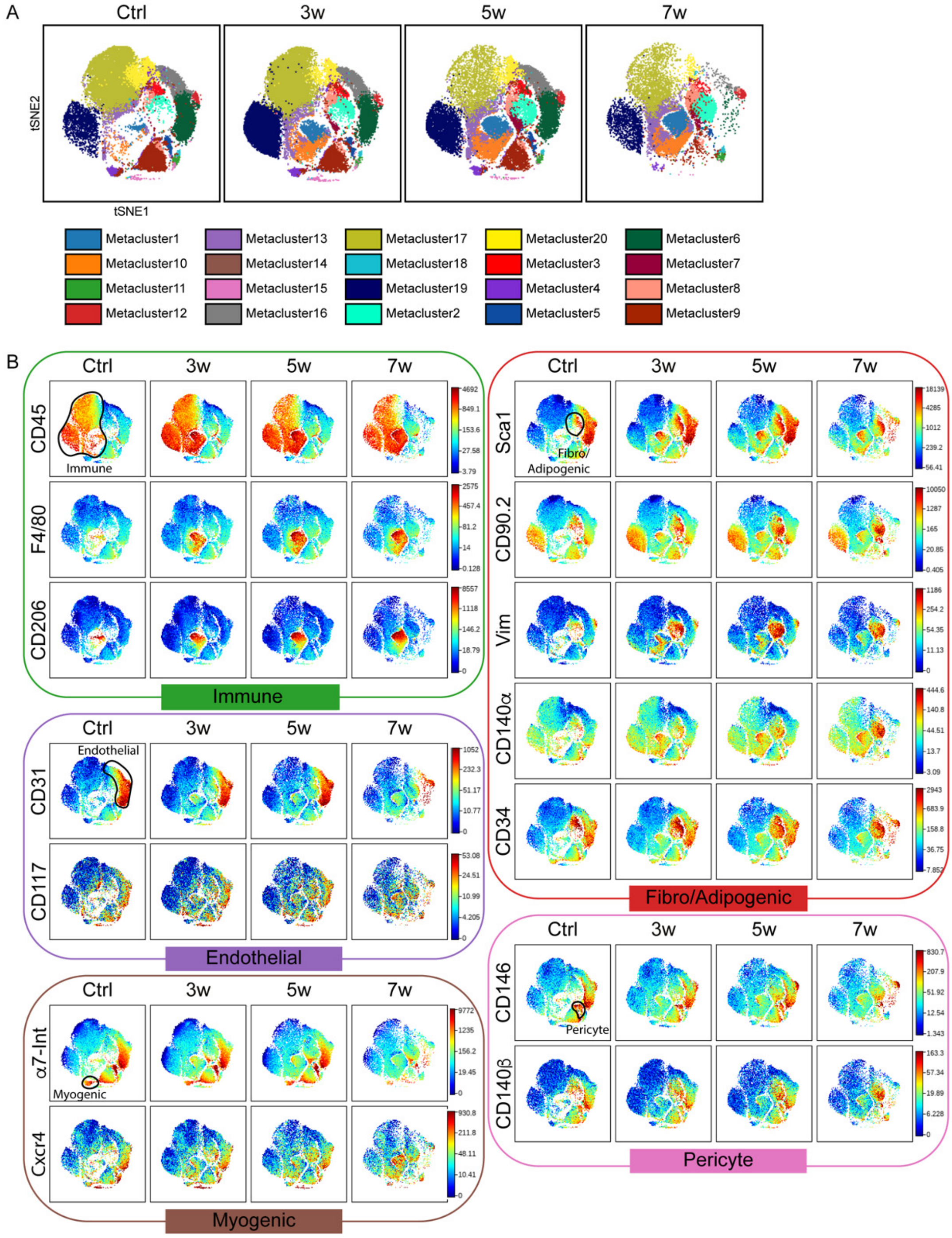

Supplementary Figure S1. Mass cytometry characterization of skeletal muscle cell subpopulations upon the induction of eRMS tumors in LSL-Kras$^{G12D/+}$;Tp53$^{Fl/Fl}$ mice. (**A**) Representative viSNE maps illustrating the 20 metaclusters created by the FlowSOM algorithm by analyzing Ctrl, three, five and seven week infected mice. (**B**) The multiparametric mass cytometry data of mononuclear cells isolated from Ctrl and Ad-Cre infected hind limb muscles of LSL-Kras$^{G12D/+}$;Tp53$^{Fl/Fl}$ mice were analyzed with the tSNE algorithm. The resulting representative viSNE maps are here colored according to the expression levels of the indicated markers (blue: low expression; red: high expression). Maps colored according to the expression of cell type specific markers are grouped (Immune, Endothelial, Myogenic, Fibro/Adipogenic and Pericyte).

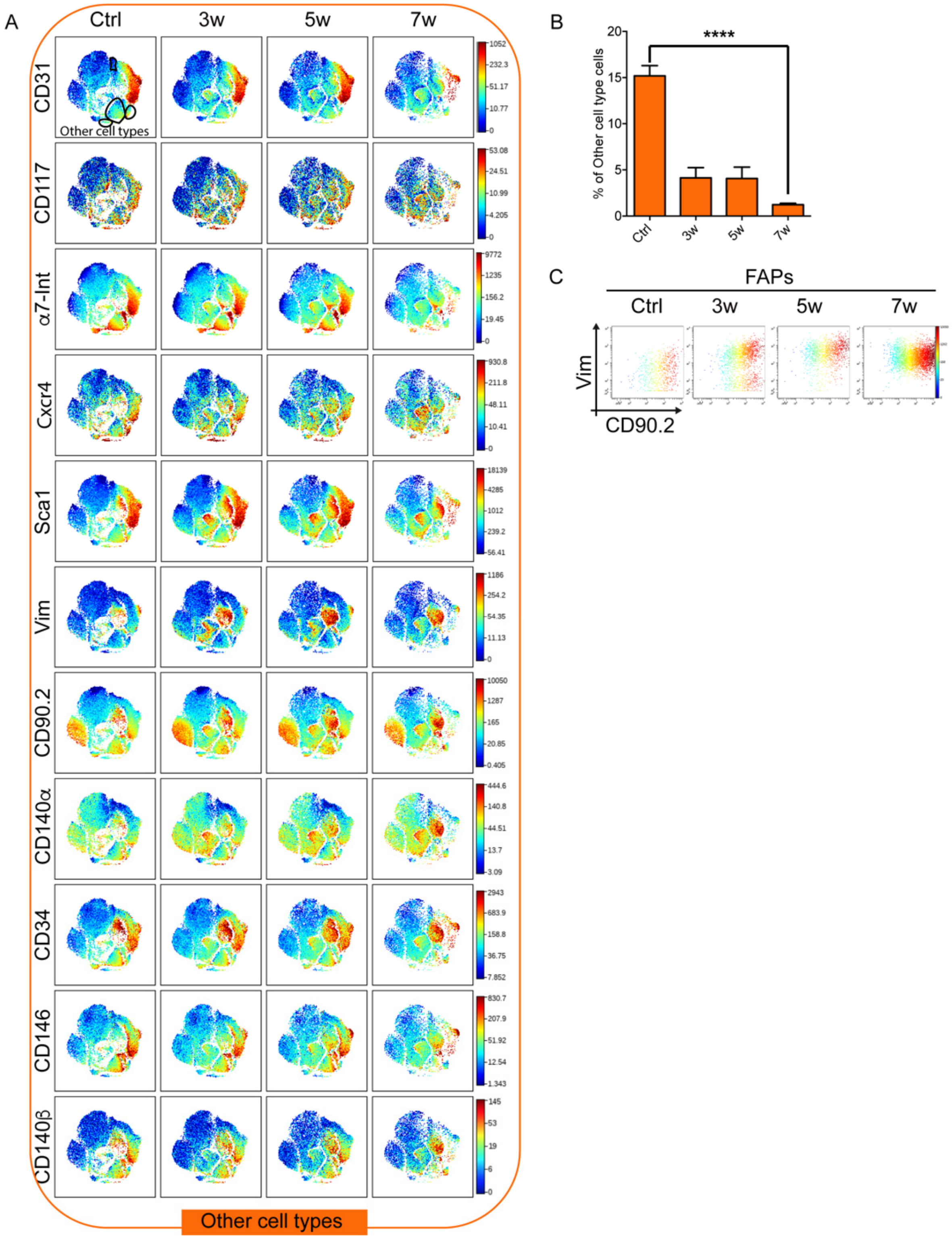

Supplementary Figure S2. Mass-cytometry characterization of "Other cell types" and of FAPs. (**A**) viSNE maps with the cell populations (circled in black) classified as "Other cell types" and showing the expression of the indicated marker (red, high expression; blue, low expression) three, five and seven weeks upon Ad-Cre in vivo infection. (**B**) Bar plots showing the relative abundance of the cell population showed in A analyzed at different time points upon Ad-Cre infection. Cell percentage was assessed on the total number of cells in each sample ($n = 3$). The statistical significance was estimated by One-way ANOVA. All data are represented as mean ± SEM and the statistical significance is defined as **** $p < 0.0001$. (**C**) The plots represent the expression of Vimentin and CD90.2 surface markers in the FAP cell population three, five and seven weeks upon Ad-Cre in vivo infection and is colored according to the expression of CD90.2.

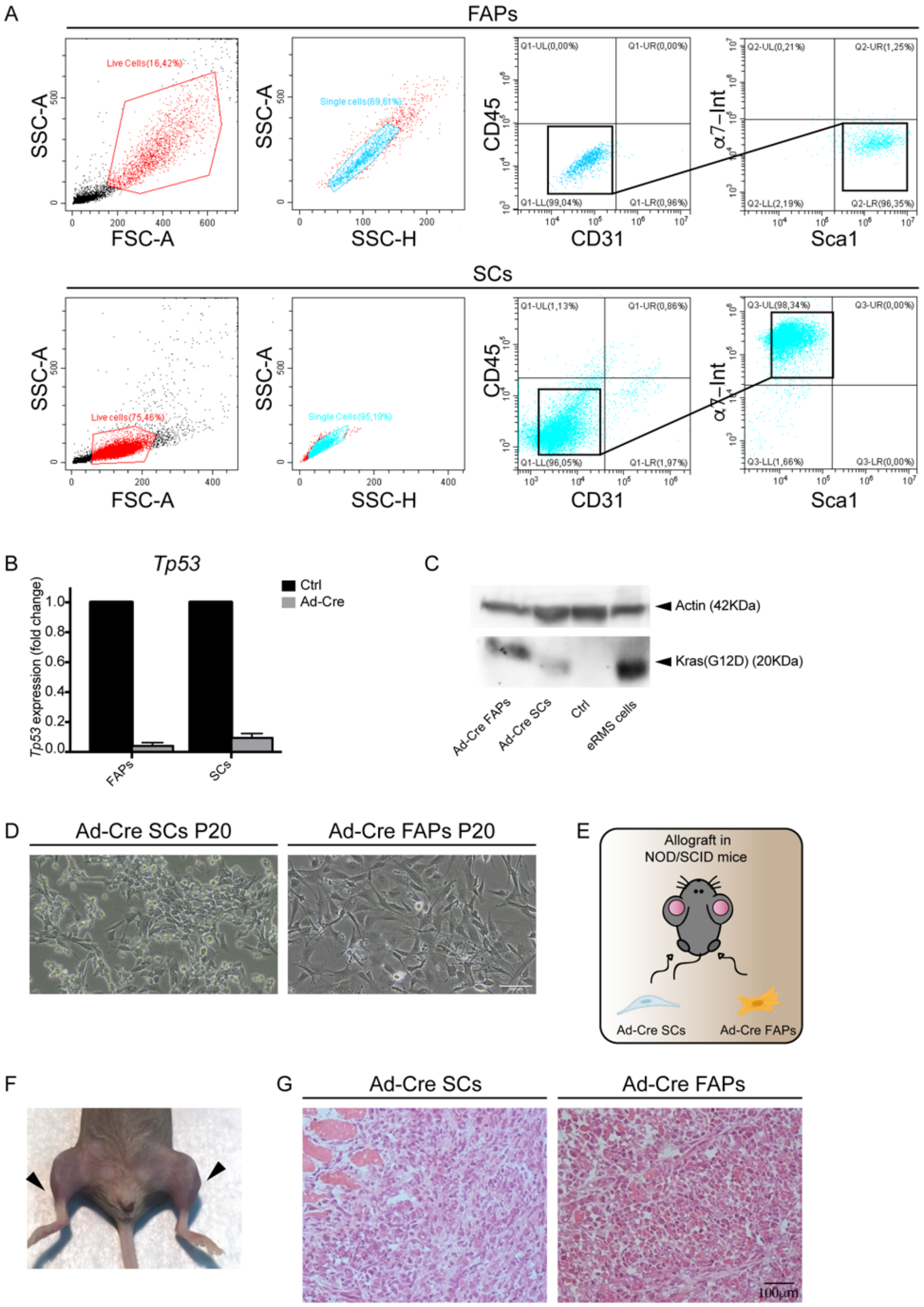

Supplementary Figure S3. Characterization of FAP and SC cells isolated from LSL-Kras$^{G12D/+}$;Tp53$^{Fl/Fl}$ conditional mice and injected into NOD/SCID mice after *in vitro* infection with Ad-Cre. (**A**) Flow cytometry analysis of the expression of CD45, CD31, Sca1 and α7-Integrin antigens in freshly isolated FAPs and SCs. Dot plots representing the gating strategy used to identify Sca1 positive FAPs (upper) and α7-Integrin positive SCs (lower). (**B**) Real-Time PCR analysis of *Tp*53 gene in FAPs and SCs three days after the Ad-Cre infection. Fold change was calculated using the $2^{-\Delta\Delta Ct}$ method and by comparison with Ctrl cells. (**C**) Representative western blot showing the constitutive expression of Kras(G12D) upon Ad-Cre infection. Actin was used as loading control. (**D**) Brightfield representative images of Ad-Cre SCs and Ad-Cre FAPs at passage 20 (P20). (**E**) Schematic illustration of in vivo inoculation of Ad-Cre SCs and Ad-Cre FAPs in NOD/SCID mice. (**F**) Images of tumors (indicated by arrows) developed in NOD/SCID mice 3.5 weeks from the inoculation of 1 x 10^5 Ad-Cre FAPs and Ad-Cre SCs in the right and left hind limb, respectively. (**G**) Representative H&E staining of tumor tissues derived from NOD/SCID mice after Ad-Cre FAP and Ad-Cre SC inoculation. (**D, G**) Scale bar 100 μm.

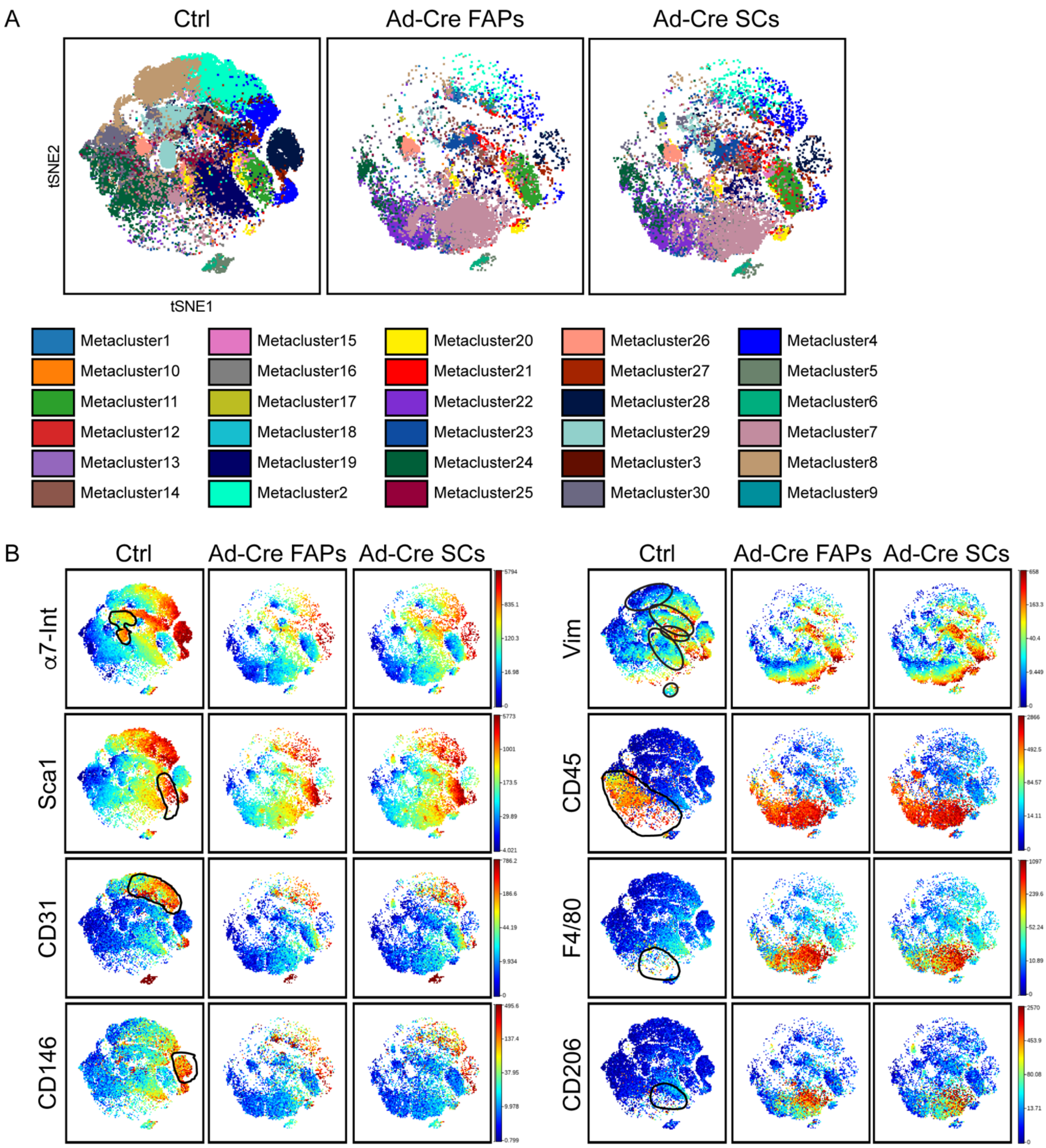

Supplementary Figure S4. Mass-cytometry characterization of allograft tumors generated upon the inoculation of Ad-Cre FAPs and Ad-Cre SCs in NOD/SCID mice. (**A**) Representative viSNE maps illustrating the 30 metaclusters created by FlowSOM algorithm by analyzing Ctrl and Ad-Cre FAP and Ad-Cre generated tumors. (**B**) The resulting representative viSNE maps are here colored according to the expression levels of the indicated markers (blue: low expression; red: high expression of the selected marker). Here are reported only the main markers used to discriminate the different cell types that were identified.

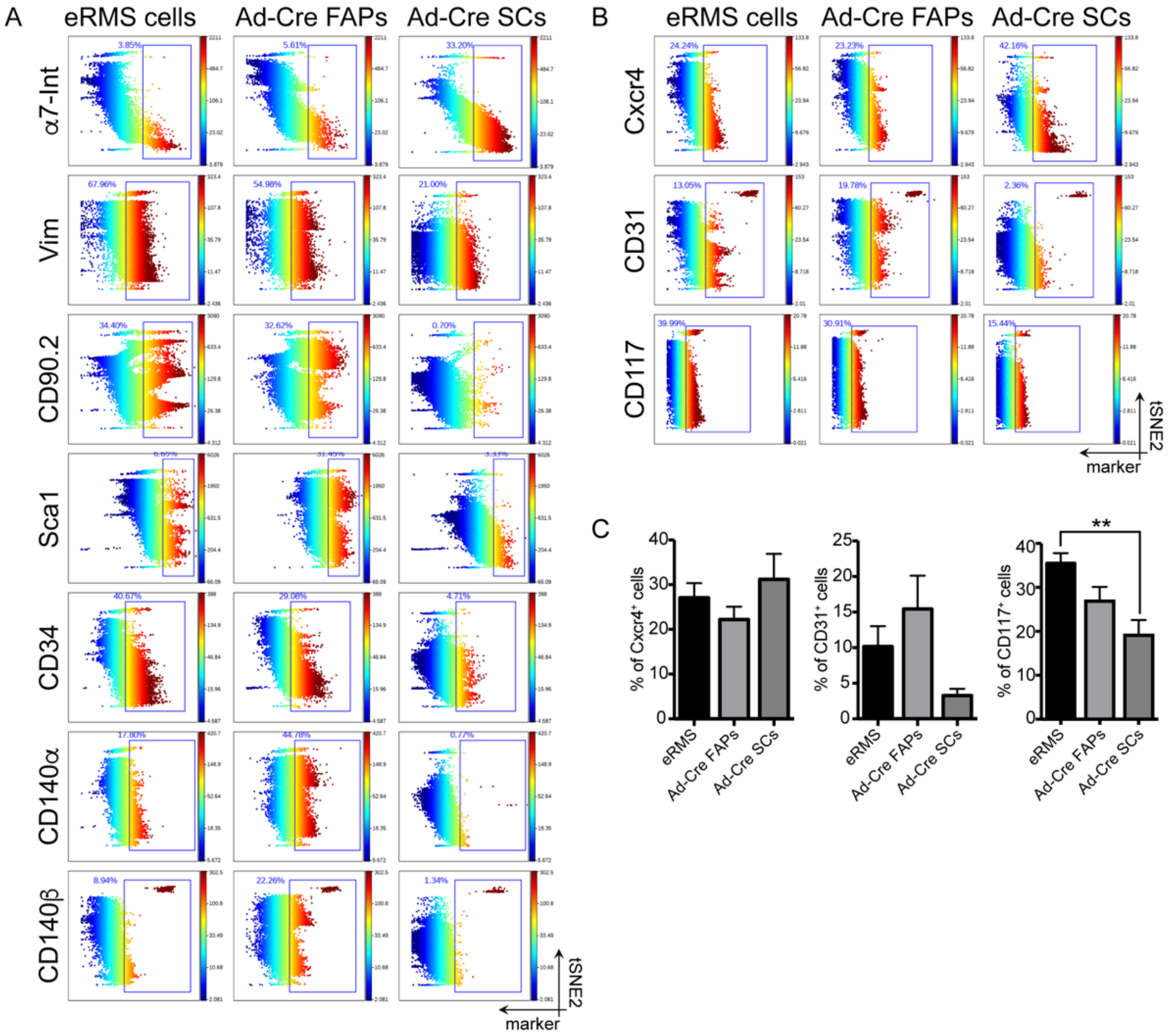

Supplementary Figure S5. Phenotypic comparison between Ad-Cre infected FAPs and Ad-Cre SCs and eRMS cells isolated from in vivo generated tumors. (**A**, **B**) Representative maps showing the gating strategy that was used to identify the abundance of the population of cells expressing the indicated marker. (**C**) Bar plots quantitate the percentage of cells positive for the expression of the indicated marker as explained in A, B. Cell percentages were assessed on the total number of cells in each sample ($n = 3$). The statistical significance was estimated by One-way ANOVA. All data are represented as mean ± SEM and the statistical significance is defined as ** $p < 0.01$.
